# Non-linear pharmacokinetics of penciclovir in healthy cats after single and multiple oral administration of famciclovir

**DOI:** 10.3389/fvets.2025.1695827

**Published:** 2025-12-01

**Authors:** Mengke Qu, Xiao Ke, Zhirong Zhang, Ling Zhang

**Affiliations:** 1 Key Laboratory of Drug-Targeting and Drug Delivery System of the Education Ministry, West China School of Pharmacy, Sichuan University, Chengdu, China; 2 Chengdu Kanghong Pharmaceuticals Group Co. Ltd., Chengdu, China; 3 Med-X Center for Materials, College of Polymer Science and Engineering, Sichuan University, Chengdu, China

**Keywords:** famciclovir, oral administration, feline, pharmacokinetics, absolute bioavailability

## Abstract

**Introduction:**

Feline Infectious Rhinobronchitis (FVR) is a common and serious infectious upper respiratory disease. Famciclovir, an antiviral prodrug initially developed for human herpesviruses, demonstrates significant therapeutic efficacy in cats with FVR caused by feline herpesvirus type 1 (FHV-1).

**Methods:**

To determine the pharmacokinetics of famciclovir in healthy cats after single or multiple oral and intravenous administrations—40 cats in 4 different dose groups received famciclovir through a single oral dose, while 10 cats received famciclovir every 12 h for 14 days, and another 10 cats received a single intravenous dose of penciclovir—in three different phases. At the predetermined time points, blood samples were collected through the radial vein of the cat. The blood samples were analyzed by liquid chromatography–tandem mass spectrometry to detect the concentration of penciclovir in cat plasma. The pharmacokinetic parameters of penciclovir were calculated using a noncompartmental model.

**Results:**

After a single oral administration in cats, the absorption and exposure of famciclovir tablets also increased with the increase in dose. After multiple oral administrations of famciclovir tablets, the concentration of the drug fluctuated violently in steady state with no accumulation in the body. The absolute bioavailability of the tested cats after single oral administration of 15.625 g, 31.25, 62.5, and 93.75 mg/kg famciclovir tablets was 67.12, 33.94, 26.45, and 18.37%, respectively.

**Discussion:**

In summary, after oral administration in cats, the absorption and exposure of famciclovir tablets showed overall non-linear pharmacokinetic characteristics. This study provides a scientific basis for the clinical dosage and duration of treatment of FVR with famciclovir tablets.

## 1 Introduction

Feline Infectious Rhinobronchitis (FVR) is a common and serious infectious upper respiratory disease, which may be fatal, especially in young kittens. FVR has an estimated seroprevalence in feline populations ranging 50–97%. The mortality rate of untreated FVR kittens can reach over 70%, and the mortality rate of untreated FVR adult cats is about 20–30% (1–5).

Feline herpesvirus type 1 (FHV-1) is believed to cause FVR. FHV-1 is an enveloped double-stranded DNA virus characterized by a glycoprotein-embedded lipid bilayer, which mediates host cell attachment and entry. Its genome exhibits strict species specificity, exclusively infecting domestic cats (*Felis catus*) and other felids, with no documented cross-species transmission to humans or canines (6). Following primary exposure, FHV-1 establishes lifelong neural latency in at least 80% of cats, and periods of viral reactivation occur throughout life in many of these cats during stress, disease, or immune decline (7). Vaccination displays a preventive effect to some extent, but vaccinated cats may still be infected with FHV-1 and become chronic carriers. The focus of FVR treatment is to reduce the frequency of recurrence and alleviate the severity of onset (8–10).

No antiviral drugs have yet been approved worldwide for the specific treatment of FHV-1 infections in cats. However, it was reported that the antiviral drug famciclovir—originally developed for human herpesvirus infection—has shown good therapeutic effects in cats. Famciclovir can significantly alleviate clinical symptoms of naturally occurring FHV-1 infection in cats and reduce the discharge of viruses from infected cat secretions (11–13). Oral administration of 30 and 90 mg/kg famciclovir twice a day for 7 consecutive days has been proven to be effective in treating spontaneous FVR cats; single oral administration of 125 mg/kg (16–52) and 500 mg/kg (92–227) to each spontaneous FVR cat is ineffective (14, 15). Oral administration of 90 mg/kg twice a day for 14 days has been effective in improving ophthalmic symptoms in cats with experimental ocular FHV-1 epithelial infection (16). Oral administration of famciclovir 90 mg/kg twice a day for up to 21 days is effective in treating spontaneous mixed upper respiratory tract diseases in cats with FHV-1 infections (17). When 3 times a day for 21 days, famciclovir improved the results of systemic, ophthalmic, clinical pathological, virological, and histological variables in cats experimentally infected with FHV-1 (18).

Although studies have shown that the use of local antiviral drugs (Ganciclovir Eye Drops) could also partially alleviate ophthalmic symptoms of FVR cats (19, 20), the oral treatment with famciclovir is considered more convenient and more effective. This is because, along the progression of the FHV-1 virus infection, it also causes in addition to symptoms in the eyes and nose, it also causing, along with the progression of the FHV-1 virus infection, it also causes diseases in other parts of the body, such as fever, cough, oral ulcers, and skin ulcers. Therefore, systemic administration is more effective for FVR treatment (18, 21).

Famciclovir is a second-generation open-loop nucleoside antiviral drug. As a prodrug, it is rapidly absorbed orally and undergoes a deacetylation reaction to convert to the main active form, penciclovir (22). Following oral administration, famciclovir undergoes rapid and extensive metabolism to penciclovir, with little to no parent compound detectable in plasma or urine. The absolute bioavailability of famciclovir is 77 ± 8% in humans (23). Unlike humans, cats exhibit poor oral absorption of famciclovir, with significant individual differences. This may be mainly due to the extremely low activity of cat liver aldehyde oxidase, which results in low famciclovir-to-penciclovir conversion (24).

It has been shown that when cats are orally given 62.5 mg (9–18 mg/kg) famciclovir, the maximum concentration (*C*_max_) of penciclovir reaches 350 ± 180 ng/mL (25). When administered 40 mg/kg famciclovir, the *C*_max_ of penciclovir in plasma increases 4 times. When the dose reached 90 mg/kg, there was no corresponding increase in the *C*_max_ of penciclovir in plasma, suggesting that it may be due to metabolic saturation causing non-linear pharmacokinetic characteristics within this dose range. When taken orally at 40 mg/kg, the absolute bioavailability is 12.5 ± 3.0%, and when taken orally at 90 mg/kg, the absolute bioavailability is 7.0 ± 1.8% (26).

The above studies indicate that the pharmacokinetics of oral famciclovir in cats are complex, with a phenomenon of metabolic saturation. The conversion of famciclovir to penciclovir occurs through the intermediate 6-deoxypenciclovir (BRL42359). Studies have reported that the conversion of BRL42359 to penciclovir in cats is the rate-limiting step, which may be a possible reason for the non-linear pharmacokinetics of famciclovir in cats. BRL42359 is a non-toxic and inactive intermediate; therefore, when studying the relationship between the pharmacokinetics and clinical dosage of famciclovir after oral administration in cats, the pharmacokinetics of the active product, penciclovir, after administration should still be examined (24).

At present, the effectiveness of the human drug famciclovir in treating FVR has been confirmed. Although several pharmacokinetic studies of penciclovir have been conducted in cats following oral administration of famciclovir, the number of cats participating in these experiments is relatively small, and the administration of different doses has not been conducted in parallel studies. Additionally, there has been no pharmacokinetic study of multiple administrations. In practice, veterinarians empirically prescribe famciclovir tablets at 30–90 mg/kg every 12 h for 14 days; therefore, the detailed pharmacokinetic data containing the above dosage in cats need to be determined. Therefore, we conducted parallel experiments to investigate the pharmacokinetic characteristics in more cats after single and multiple oral administration of famciclovir tablets and intravenous injection of penciclovir.

## 2 Materials and methods

### 2.1 Animals

Sixty sexually intact experimental British shorthair cats, purchased from Xinuogu Biotechnology Co., Ltd., Jiangsu, China, were included, with half male and half female (no pregnant), aged 12.25 ± 0.69 months, and body weights ranging from 1 to 4 kg. The experimental cats were fed the same standard cat food (KERES Full Price Adult Cat Food, LOT: 20240420, China) for 7 days before administration to adapt to the study protocol and continued to be fed the same food during the induction. A cat was fed approximately 75 g of feed each time, twice a day. The cats took filtered drinking water freely from the water box. Complete physical examination and routine laboratory testing were performed on the cats to assess their general health: good mental state and nutritional status, no external injuries, respiration (15–30 times/min), heart rate (120–140 times/min), rectal temperature (38.0–39.2 °C), systolic blood pressure (120–140 mm Hg), neither diseases nor pathological conditions interfering with the experiment.

The animal study protocol (Protocol Number FXLW-M-PK-01) was approved by the Institutional Review Board of Beijing Sun-Demei Pharmaceutical Technology Co., Ltd., Beijing, China.

### 2.2 Experimental protocol

The study was divided into three phases. Phase I consisted of a single oral administration; a balanced crossover design was used with 40 cats randomly assigned (half male and half female) to 4 dose groups (15.625, 31.25, 62.5, and 93.75 mg/kg famciclovir). Phase II consisted of a multiple-dose trial of famciclovir given orally every 12 h (*n* = 10 cats, half male and half female; 62.5 mg/kg) for 14 days. Phase III consisted of a single intravenous administration of 10 mg/kg penciclovir.

Famciclovir tablets (125 mg, purchased as a human medicine) were carefully cut into four equal portions with a pill cutter. Before a single oral administration, animals were fasted for 12 h and then fed uniformly 4 h after receiving the medication. During multiple oral administrations, animals should be fasted for 8 h before each administration and fed uniformly 4 h after administration of the medication. The oral administration operation is as follows: raise the cat’s head and gently press the upper lip on both sides into the mouth, starting from the back of the cat’s nose, to encourage the cat to open its mouth. Use the middle and ring fingers to press against the corners of the mouth to help open it. Then use a special dispenser to hold the medicine and deliver it into the throat. Quickly close the mouth and lightly tap the lower jaw to let it dissolve. When a cat licks its nose with its tongue, it indicates that it has ingested the medicine. During the medication period, observe the cat’s mouth and nose for any changes in condition.

In phase III, another ten cats (half male and half female) received 10 mg/kg penciclovir via radial vein infusion (1 h). Before intravenous administration, animals were fasted for 12 h and then fed uniformly 4 h after administration. The preparation of penciclovir injection requires a sterile environment during the preparation process. Dissolve in physiological saline (0.9% NaCl) solution, then adjust the pH of the solution to 11.0 (25 °C) with sodium hydroxide; dilute with physiological saline to the final administration concentration of 2 mg/mL, filter through a 0.22-μm filter membrane, and seal in a sterile bottle for later use. After sterile testing, it can be used for intravenous administration in animals.

### 2.3 Blood collection

At each of the following time points, 0.6–1 mL of blood sample is collected through the radial vein of the cat and placed in a blood collection tube containing anticoagulant dipotassium ethylenediaminetetraacetic acid (EDTA-K_2_). The specific procedure of blood collection is as follows: with the cat’s forelimbs extended and stabilized, we bent the cat’s wrist joint to tension its radial vein. After locating the radial vein (1–2 cm above the wrist joint on the medial side of the radius), we disinfected the skin in a circular motion from the inside out, centered around the blood collection point, using a 75% alcohol cotton ball. The bevel of the needle was placed upward, forming a slight angle of 10°–20° with the skin, aiming at the radial vein and quickly and smoothly piercing the skin. Once the needle tip entered the vein with an insertion depth of only 2–3 mm, the syringe plunger was gently withdrawn, allowing a small amount of blood to flow into the needle tube. Then we pressed gently on the puncture site with a dry cotton ball and quickly yet steadily withdrew the needle, immediately applying firm pressure to the blood collection site for 1–2 min until complete hemostasis was confirmed. Repeat the above procedure at each time point until the blood collection is complete.

#### 2.3.1 Phase I

Collect blood samples at 0 (before the administration), 15, and 30 min; and 1.0, 1.5, 2.0, 3.0, 4.0, 5.0, 6.0, 8.0, 10.0, 12.0, and 36.0 h. The blood collection time range is 10–30 min ± 10 s; 30 ~ 1 h ± 30 s; 1–2 h ± 2 min; 2 ~ 10 h ± 10 min; and 12.0 h and later ± 30 min.

#### 2.3.2 Phase II

Day 1: Collect blood samples within 30 min before the first administration and after the first administration at 15 and 30 min and 1.0, 1.5, 2.0, 3.0, 4.0, 5.0, 6.0, 8.0, 10.0, and 12.0; Day 13: Collect blood samples within 30 min before the first and the second administration; Day 14: Collect blood samples within 30 min before the first administration and after the first administration at 15 and 30 min and 1.0, 1.5, 2.0, 3.0, 4.0, 5.0, 6.0, 8.0, 10.0, 12.0, and 36.0 h. The blood collection time range is the same as phase I.

#### 2.3.3 Phase III

Notably, 1 h of intravenous drip administration and collect blood samples within 30 min before drip administration; 20 min and 40 min during administration; and 0 min immediately after administration; 5, 10, and 30 min, and 1.0, 1.5, 2.0, 4.0, 6.0, 8.0, and 12 h after administration.

After collecting the blood sample, it was transferred to the sampling tube by gently inverting and shaking back and forth. The labeled sample was quickly transferred to a centrifuge and centrifuged at 2,500 *g* (centrifuge set at 4 °C) for 10 min. Then 0.2 mL of plasma from each sample was transferred to the detection tube, and the remaining sample was transferred to the backup tube. The plasma sample can be temporarily stored in a −20 °C freezer and transferred to a −70 °C freezer within 15 days.

### 2.4 Famciclovir assay

The blood samples were analyzed using a validated liquid chromatography–tandem mass spectrometry (LC–MS/MS) assay technique to detect the concentration of penciclovir in cat plasma. Penciclovir D4 was simultaneously analyzed as an internal standard. The samples were pretreated using the protein precipitation method, then atomized and ionized using the ionization technology by the electrospray ionization technique. Subsequently, the sample concentration was measured in MRM mode.

Sample pretreatment method: 25 μL of the cat plasma sample was accurately measured and placed in a processing tube with 25 μL of the internal standard working solution (1,000,000 ng/mL) separately added (if the processed sample is a double blank sample, add 50% acetonitrile aqueous solution instead of the internal standard working solution). Notably, 200 μL of acetonitrile was added separately to the tube, followed by vortexing at 2,500 rpm for 10 min and thorough mixing. Then, the sample was centrifuged at high speed for 10 min at a low temperature of 2–8 °C (set at 4 °C) and 4,000 *g*. 50 μL of the supernatant was taken and added to 150 μL of 10% acetonitrile aqueous solution, followed by vortexing at 1,000 rpm for 3 min and mixing evenly. 5.0 μL of the sample was injected for LC–MS/MS analysis.

The analytical system consisted of an Xevo TQ-XS triple quadrupole mass spectrometer, Waters Corporation, Massachusetts, USA; coupled to an Acquity I-class Plus UPLC system (Waters), Waters Corporation, Massachusetts, USA. Samples were chromatographed on a Shim-pack GIST C18-AQ (3.0 × 50 mm, 3 μm) (SHIMADZU), Shimadzu Corporation, Japan. Continuous automatic injection and automatic integration methods were used for chromatogram collection and integration. Linear regression was performed between the concentration of the analyte and the response value (the ratio of the analyte to its internal standard peak area).

A liquid chromatography gradient was employed with mobile phase A consisting of 0.1% formic acid-water and mobile phase B composed of 0.1% formic acid-acetonitrile at 250 μL/min.

**Table:** 

Time (min)	%A	%B
0.00	95.0	5.0
0.30	95.0	5.0
1.00	10.0	90.0
2.00	10.0	90.0
2.10	95.0	5.0
3.00	95.0	5.0

The mass spectrometer settings were optimized as follows:

**Table:** 

Capillary (kV)		3.5	Cone gas flow (L/h)		300
Source offset (V)		30.0	Desolvation gas flow (L/h)		1,000
Source temperature (°C)		150	Collision gas flow (mt/min)		0.15
Desolvation temperature (°C)		600	Nebulizer gas flow (bar)		7.00
ID	Parent ion (m/z)	Daughter ion (m/z)	Cone (V)	Coll (eV)	Dwell (s)
PEN	254.20	152.10	30.00	15.00	0.163
PEN-IS	258.20	152.10	30.00	15.00	0.163

PEN, Penciclovir; PEN-IS, Penciclovir-D4.

### 2.5 Absolute bioavailability

Through WinNonlin software, using data obtained from non-compartmental analysis and performing dose normalization correction, absolute bioavailability data were obtained.

Absolute bioavailability:


$$F\%=\frac{{\mathrm{AUC}}_{\text{oral}}\times {\text{Dose}}_{\mathrm{iv}}\times {\mathrm{MW}}_{\mathrm{FCV}}}{{\mathrm{AUC}}_{\mathrm{iv}}\times {\text{Dose}}_{\text{oral}}\times {\mathrm{MW}}_{\mathrm{PCV}}}\times 100\%$$

whereF% is absolute bioavailability, AUC is the area under the drug time curve, MW_FCV_ is the molecular weight of famciclovir (321.33 g/mol), MW_PCV_ is the molecular weight of penciclovir (253.26 g/mol), Dose_iv_ is 10 mg/kg penciclovir, and Dose_oral_ is the dosage of famciclovir.

### 2.6 Pharmacokinetic and statistical analysis

Using a pharmacokinetic concentration set, the pharmacokinetic parameters of penciclovir in the plasma of each animal were calculated using a non-compartmental model. Using a pharmacokinetic parameter set, calculate the arithmetic mean, standard deviation, relative standard deviation, geometric mean, median, maximum, and minimum values of each parameter, and summarize and describe the results of pharmacokinetic parameters (PK) analysis. If the AUC_%Extrap_ of the test cat were greater than 20%, descriptive statistical analysis would not be performed on AUC_0−∞_, elimination half-life (*t*_1/2_), and AUC_%Extrap_. The PK and their definitions for single-dose studies were detailed in Supplementary Table S1, while those for multiple-dose studies were detailed in Supplementary Table S2.

We conducted a significance test analysis (*t*-test) on the dose factors of the parameters (*C*_max_, AUC_0−*t*_, and AUC_0−∞_) in test cats with single oral administration. Additionally, we performed a significance test analysis (Mann–Whitney *U* test) on the gender factors of various pharmacokinetic parameters in all test cats.

## 3 Results

### 3.1 Drug administration

After administration, all animals experienced no vomiting or other adverse symptoms, indicating good medication adherence. During the experiment, the mental state, nutritional status, fur condition, urination, and defecation of the test animals were observed daily. The cats had no stress reactions, normal feeding behavior, and good health. During the feeding and administration period, the animals consumed approximately 150 g of feed per day, with no decrease in their food intake observed.

Sixty animals involved in the experiment, and none dropped out or was excluded. There was neither an animal reused after the washout period for different drug dosages nor used after any other drug research. All 60 animals were enrolled in this study protocol and were completed and included in the statistical analysis.

### 3.2 Famciclovir assay performance

The analytical method established in this study was linearly good (*r* ≥ 0.99) in the concentration range of 50.0–10,000 ng/mL; the average recovery rate for the three levels of addition at 150, 4,000, and 8,000 ng/mL was 88.07, 91.40, and 89.99%, respectively, with relative standard deviation less than 10%. The lower quantitative limit was 50.0 ng/mL.

The validation results of this method meet the acceptance criteria for standard curves, selectivity and specificity, matrix effect, residue, precision and accuracy, quantitative lower limits, extraction recovery rate, dilution reliability, analytical batch capacity, stability, and reproducibility of post-treatment sample re-entry specified in this project’s methodology validation program. The method is specific, sensitive, with good precision and accuracy, and has been proven suitable for determining penciclovir concentrations in cat plasma matrices containing EDTA-K_2_ anticoagulant with a sample dose of 25 μL and a linear range of 50.0–10,000 ng/mL for penciclovir.

### 3.3 Pharmacokinetic analysis

After a single oral administration in cats, the absorption and exposure of famciclovir tablets increased with the dose, exhibiting overall non-linear pharmacokinetic characteristics.

**Figure 1:**
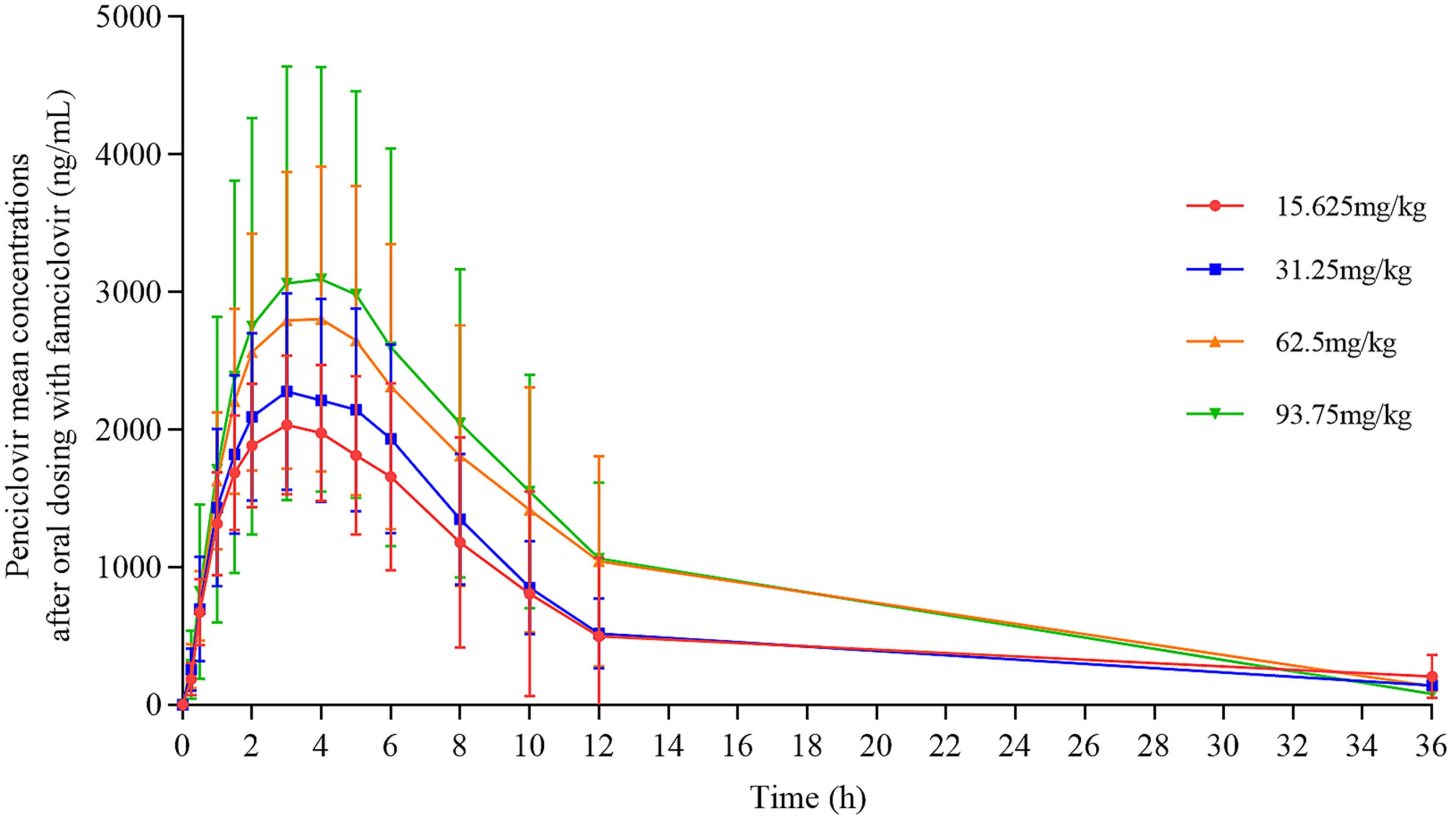
Mean plasma concentration–time curve of penciclovir in cats after oral administration of famciclovir.

Four different doses of famciclovir tablets were administered orally to cats in separate groups, with 10 cats in each group. Blood samples were taken at designated time points to measure the drug concentration (Figure 1). As the dosage increases, the *C*_max_ value increases, while the time to peak (*T*_max_) remains relatively consistent. The plasma concentration time curves of penciclovir of each individual cat in each dose group have been shown (Supplementary Figures S1–S4).

The PK of penciclovir in the plasma of the tested cats after single oral administration of 15.625, 31.25, 62.5, and 93.75 mg/kg famciclovir tablets were shown (Table 1), including *C*_max_, *T*_max_, AUC_0−*t*_, AUC_0−∞_, *t*_1/2_, mean retention time (MRT), clearance rate (Cl/F), elimination rate constant (*λ*_z_), apparent volume of distribution (*V*z/*F*), AUC_%, Extrap_obs_. There were significant differences in the statistical comparison of C_max_, AUC_0-t_, and AUC_0−∞_ between different dosage groups (Supplementary Table S3). There were significant differences in *C*_max_, AUC_0−*t*_, and AUC_0−∞_ between the 15.625 mg/kg dose group and the other three dose groups. There were significant differences in *C*_max_, AUC_0−*t*_, and AUC_0−∞_ between the 31.25 and 93.75 mg/kg dose groups, but only *C*_max_ showed significant differences compared to the 31.25 and 62.5 mg/kg dose groups. There were no significant differences in *C*_max_, AUC_0−*t*_, and AUC_0−∞_ between the 62.5 and 93.75 mg/kg dose groups.

**Table 1:** The PK of penciclovir in the plasma of the tested cats after single oral administration (mean ± SD).

PK	Unit	15.625 mg/kg	31.25 mg/kg	62.5 mg/kg	93.75 mg/kg
*λ*_z_	1/h	0.2800 ± 0.10	0.2600 ± 0.15	0.1500 ± 0.07	0.1500 ± 0.05
*t*_1/2_	h	3.25 ± 2.48	3.84 ± 2.87	5.82 ± 3.39	5.21 ± 1.64
*T*_max_	h	3.45 ± 1.60	3.25 ± 0.79	3.60 ± 1.20	3.40 ± 1.10
*C*_max_	ng/mL	2,170 ± 560	2,340 ± 690	2,890 ± 1,100	3,290 ± 1,500
AUC_0−*t*_	h ng mL^−1^	20,229.38 ± 14,437.12	18,833.78 ± 5,514.35	26,871.53 ± 14,770.37	29,499.80 ± 14,056.33
AUC_0−∞_	h ng mL^−1^	21,347.13 ± 15,238.74	21,589.18 ± 6,209.66	33,645.94 ± 18,437.41	35,063.68 ± 15,723.11
AUC_%Extrap_obs_	%	5.06 ± 2.17	11.93 ± 10.88	18.30 ± 12.90	15.34 ± 12.73
*V*z/*F*__obs_	L	7.55 ± 2.12	17.93 ± 11.35	41.93 ± 19.84	50.24 ± 21.23
Cl/F__obs_	L/h	2.11 ± 1.00	3.58 ± 1.41	5.90 ± 3.18	6.90 ± 2.69
MRTlast	h	5.80 ± 2.34	5.63 ± 1.49	6.21 ± 1.81	6.51 ± 1.72

**Figure 2:**
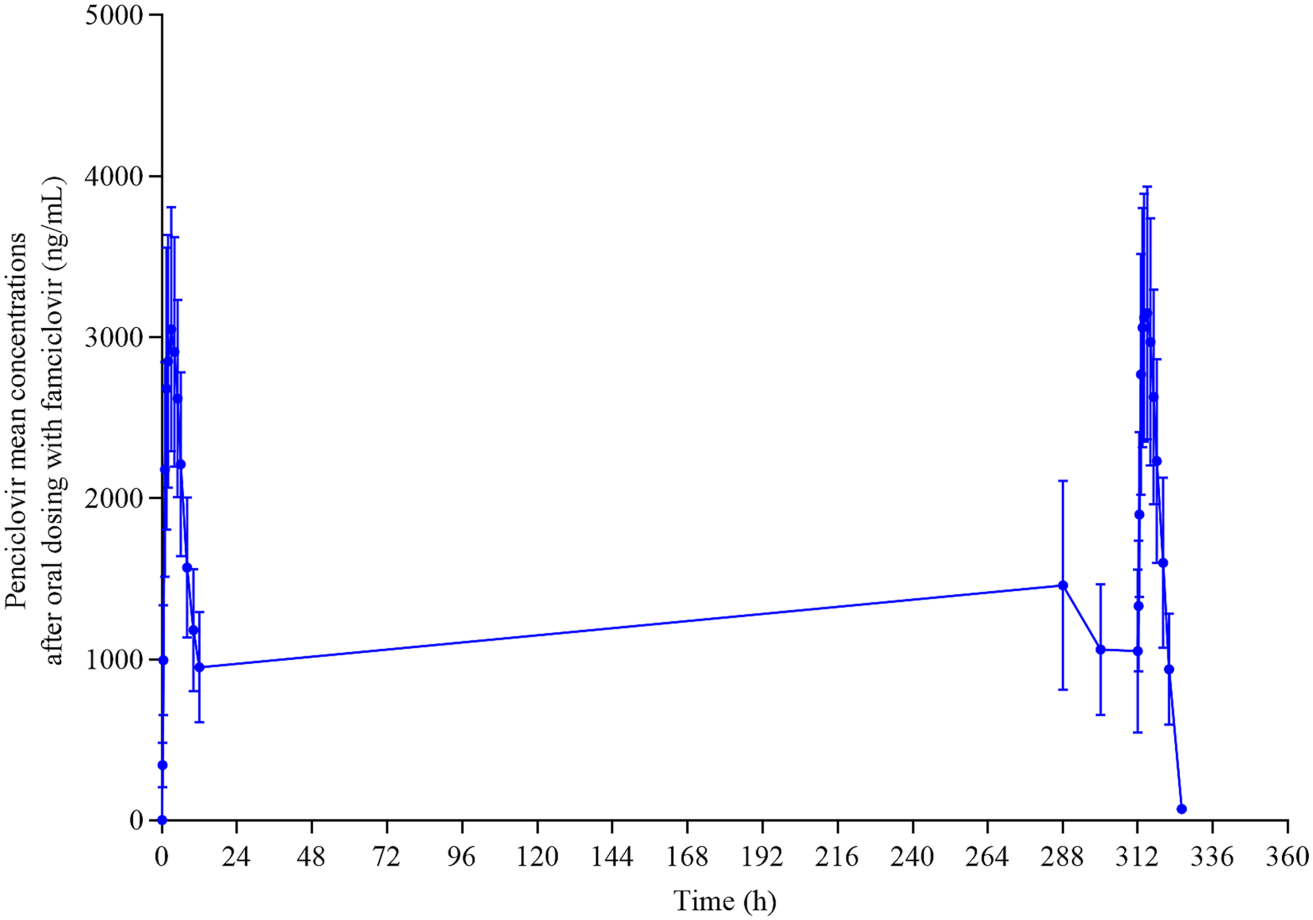
Mean plasma concentration–time curve of penciclovir in cats after continuous oral administration of famciclovir.

After multiple oral administrations of 62.5 mg/kg famciclovir tablets twice daily for 14 consecutive days to reach steady state (Figure 2), *T*_max,ss_ of penciclovir in the plasma of the test cats were 2.6 ± 0.81 h. *C*_max,ss_ was 3,197 ± 782.97 ng/mL, *C*_min,ss_ was 1,047 ± 508.44 ng/mL, *C*_av,ss_ was 2,100.31 ± 562.54 ng/mL, and volatility coefficient (DF) was 105.69 ± 0.25%, indicating that the concentration of the drug fluctuating violently in steady state with a peak valley concentration difference close to 1.06 times the average concentration, which was consistent with the characteristics of rapid release formulations. Accumulation factors (Rac_*C*_max_ and Rac_AUC) were 1.06 ± 0.23 and 1.09 ± 0.21, respectively, with both accumulation factors close to 1, indicating that the drug had almost no accumulation in the body, was rapidly cleared, and could be completely excreted from the body within the dosing interval. The PK of penciclovir in the plasma of the tested cats after a multiple-dose trial of 62.5 mg/kg famciclovir tablets given every 12 h for 14 days has been shown (Table 2).

**Table 2:** The PK of penciclovir in the plasma of the tested cats after a multiple-dose trial (Mean ± SD).

PK	Unit	62.5 mg/kg
*t*_1/2_	h	5.79 ± 1.77
*T*_max, D1_	h	2.9 ± 0.67
*C*_max, D1_	ng/mL	3,072 ± 763.4
*T*_max, ss_	h	2.6 ± 0.81
*C*_max, ss_	ng/mL	3,197 ± 782.97
*C*_min, ss_	ng/mL	1,047 ± 508.44
*C*_av, ss_	ng/mL	2,100.31 ± 562.54
AUC_0–12, D1_	h ng mL^−1^	23,362.8 ± 5603.77
AUC_0–12, ss_	h ng mL^−1^	25,203.75 ± 6750.48
*V*_ss_/*F*	L	46.71 ± 17.33
CL_ss_/*F*	L/h	5.91 ± 2.13
DF	%	105.69 ± 0.25
Rac_C_max_	/	1.06 ± 0.23
Rac_AUC	/	1.09 ± 0.21

**Figure 3:**
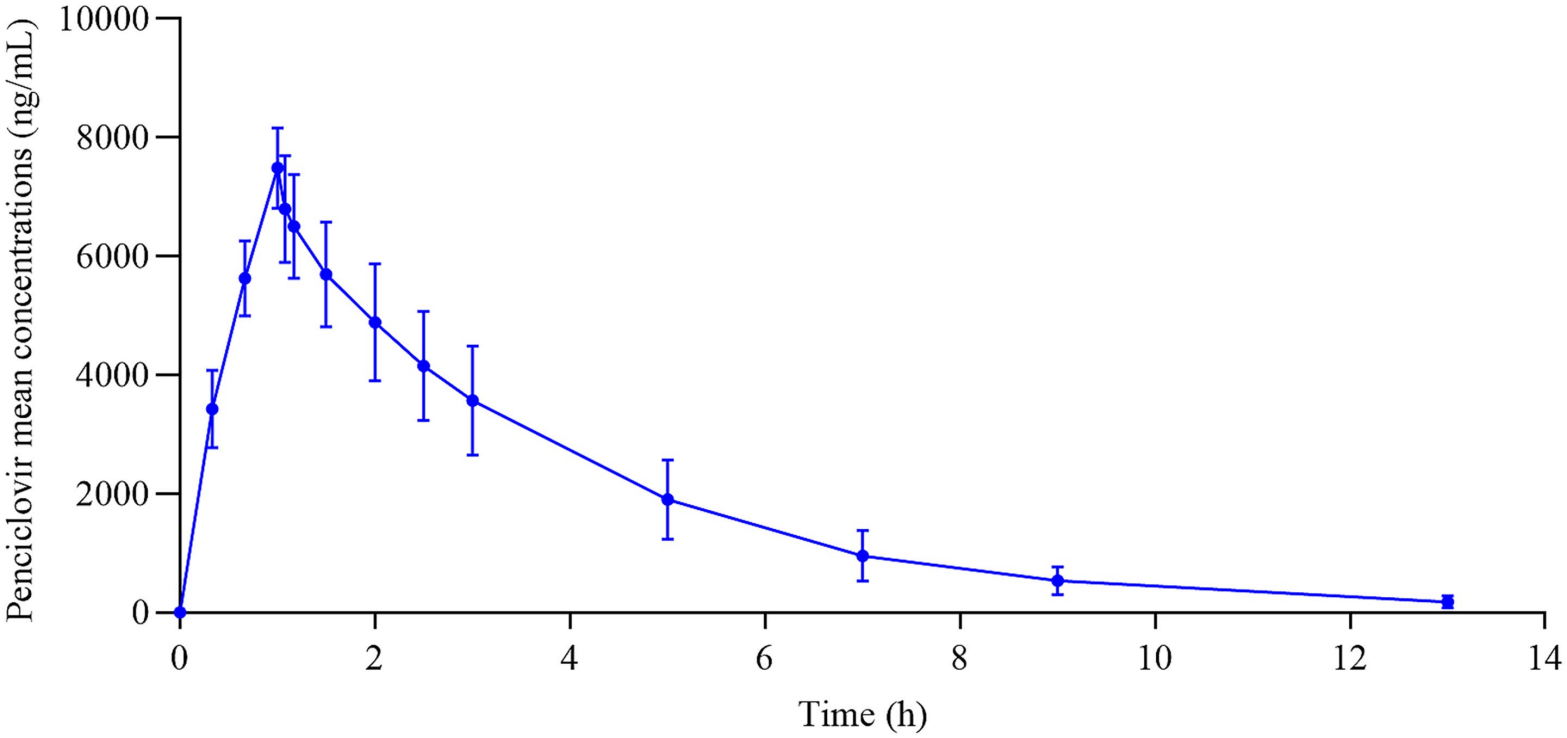
Mean plasma concentration–time curve of penciclovir after intravenous infusion of 10 mg/kg penciclovir in the tested cats.

After intravenous infusion of 10 mg/kg penciclovir, *T*_max_, *C*_max_, AUC_0−*t*_, and AUC_0−∞_ of penciclovir in the plasma of the tested cats were 1.017 ± 0.035 h, 7,572 ± 557.97 ng/mL, 25266.77 ± 5299.71 h ng mL^−1^, and 25823.71 ± 5514.8 h ng mL^−1^, respectively. Intravenous administration directly enters the systemic circulation without an absorption barrier; therefore, its AUC reflects the ideal state of 100% utilization of the drug (Figure 3). By comparing the AUC of oral administration, the degree of loss caused by incomplete absorption or first-pass elimination of the drug can be quantified.

The PK of penciclovir in the plasma of the tested cats that received 10 mg/kg penciclovir via a single intravenous administration has been shown (Table 3).

**Table 3:** The PK of penciclovir in the plasma of the tested cats received penciclovir after a single intravenous administration (mean ± SD).

PK	Unit	10 mg/kg
*λ*_z_	1/h	0.366 ± 0.105
*t*_1/2_	h	2.03 ± 0.54
*T*_max_	h	1.017 ± 0.035
*C*_max_	ng/mL	7,572 ± 557.97
AUC_0−*t*_	h·ng·mL^−1^	25,266.77 ± 5,299.71
AUC_0−∞_	h·ng·mL^−1^	25,823.71 ± 5,514.8
AUC_%Extrap_	%	2.07 ± 1.33
*V*z/*F*	L	3.33 ± 0.68
Cl/F	L/h	1.21 ± 0.43
MRT_0−*t*_	h	2.61 ± 0.59
MRT_0−∞_	h	2.86 ± 0.73

The significance test analysis of gender factors on the pharmacokinetic parameters of the test cats has been shown (Supplementary Table S4). Except for the significant difference (*p* = 0.047) of MRT_0−*t*_ between female and male cats in the single-dose study at a dose of 62.5 mg/kg and the significant difference (*p* = 0.016) of *V*z/*F* between female and male cats in the intravenous injection study, there were no significant differences (*p* > 0.05) in the pharmacokinetic parameters between female and male test cats. The reason for the significant difference of MRT_0−*t*_ in the single-dose study at a dose of 62.5 mg/kg was that cats numbers 1 and 5 had detectable concentrations at 36 h, resulting in a higher MRT_0−*t*_; the reason for the significant difference of *V*z/*F* in the intravenous injection study was that the average body weight of male cats was higher than that of female cats, which is a reasonable phenomenon. The results indicated that there were no gender differences in the pharmacokinetic parameters of famciclovir under single-dose, multiple-dose, and intravenous administration conditions.

### 3.4 Absolute bioavailability

The absolute bioavailability with dose normalization correction of penciclovir in the plasma of the tested cats after single oral administration of 15.625, 31.25, 62.5, and 93.75 famciclovir tablets was shown (Table 4). As the oral dosage increased, the *F*% of famciclovir tablets decreased and showed a negative growth trend.

**Table 4:** Absolute bioavailability with dose normalization correction results of different doses of famciclovir tablets orally administered.

Doses	15.625 mg/kg	31.25 mg/kg	62.5 mg/kg	93.75 mg/kg
*F*%	67.12%	33.94%	26.45%	18.37%

## 4 Discussion

As a prodrug, famciclovir first undergoes a deacetylation reaction after absorption in the intestinal wall, is oxidized to its main active form, penciclovir (22). Oral administration of famciclovir rarely or almost never detects the prototype drug in plasma and urine, so this study chose to detect the concentration of penciclovir in plasma to characterize the pharmacokinetic characteristics of famciclovir in the tested cats.

The oral bioavailability of penciclovir is extremely low (only about 5–10% in humans), making it difficult to use directly as an oral medication (27). Famciclovir significantly improves oral absorption rate through structural modification (diacetyl ester derivative of penciclovir) (23). Due to poor oral absorption, penciclovir is only suitable for topical or injectable use, while famciclovir, as its prodrug, is convenient for oral administration. Therefore, when examining the relative bioavailability of famciclovir tablets in felines, we used intravenous injection of penciclovir as a reference.

The stable isotope-labeled internal standard of penciclovir D_4_ has the same physicochemical properties as the analyte penciclovir, which can improve the reliability and repeatability of the detection method (28). Therefore, we selected stable isotope compounds with the same chemical structure as the analyte, penciclovir, as the internal standard for the experiment.

According to the pharmacokinetic data, different from humans, there is poor oral absorption of famciclovir in cats, which is consistent with previous research results (26). As shown in Table 4, the absolute bioavailability of famciclovir tablets decreases with increasing oral dosage, showing a negative growth trend. This may be mainly due to the extremely low activity of cat liver aldehyde oxidase, which results in poor ability to convert famciclovir to penciclovir, causing high doses of famciclovir tablets not to be completely converted to penciclovir after oral administration (24). Therefore, the pharmacokinetic process of famciclovir tablets in cats is more complex and presents non-linear pharmacokinetic characteristics.

**Figure 4:**
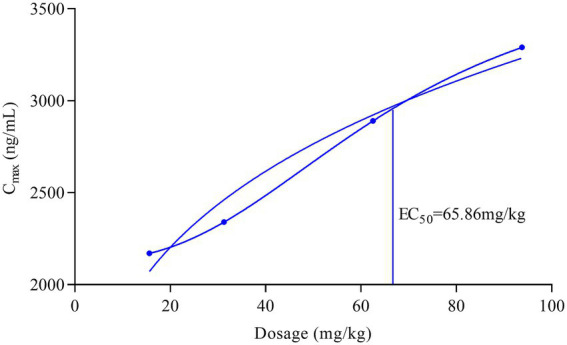
The dose-*C*_max_ response curve (sigmoid curve) of famciclovir tablets oral administration.

In this experiment, the single oral dose increased (15.625, 31.25, 62.5, 93.75 mg/kg), but *C*_max_ and AUC did not increase proportionally. Therefore, we explored the “dose–response relationship.” The dose–*C*_max_ response curve (sigmoid curve) visualizes the non-linear effect of increasing the dosage by one unit on the oral absorption pharmacokinetic characteristics of famciclovir tablets (Figure 4). Through curve simulation, the dosage [half maximal effective concentration (EC_50_)] that reaches half of the maximum drug concentration is 65.86 mg/kg, and the rate of increase in C_max_ slows down as the dosage increases. This result is consistent with the previous discussion.

The bioavailability data from this study are so different from those from previous work that they permit some interesting observations. We obtained absolute bioavailability data (the bioavailability in cats following single oral administration of 15.625, 31.25, 62.5, and 93.75 mg/kg famciclovir was 67.12, 33.94, 26.45, and 18.37%, respectively) that was higher than previously reported (the bioavailability in cats following single oral administration of 40 and 90 mg/kg famciclovir was 12.5 ± 3.0 and 7.0 ± 1.8%, respectively) (26); basically due to that, our study got much lower *C*_max_ and AUC after intravenous infusion of 10 mg/kg penciclovir but much higher *C*_max_ and AUC when giving famciclovir orally. The difference in higher *C*_max_ and AUC when giving famciclovir orally could be due to the influence of food intake. It has been shown that food may reduce the absorption of famciclovir tablets (25). *C*_max_ of penciclovir was significantly decreased by 53% with *T*_max_ from 0.9 to 2.25 h when oral administration of famciclovir was 0.5 h after ingestion of food in humans (29). Therefore, to avoid the impact of food on the pharmacokinetic characteristics of cats after oral administration, we fasted for 8 or 12 h before administration, whereas food was available at all times for cats in the previous study (26). Although fasting administration reduced the impact of food on absorption in this study, there were still significant individual differences in plasma drug concentration in the same dose group, due to the inconsistent levels of hepatic aldehyde oxidase activity among individual cats, as shown in Supplementary Figures S1–S4. The conversion ability of a few cats was significantly higher than the average level, which may be related to their individual developmental level. In this study, the test cats were of the same breed (British Shorthair cat) from the same purchased source, which differed from those cat breeds used in previous research, and we did not study the impact of varieties. So, the differences in PK may stem from variations in breed varieties, and further research and exploration are needed. Besides feeding and cat breeds, this apparent difference of *C*_max_ and AUC when giving famciclovir orally was most likely attributable to differences in sample collection time points, considering that blood was collected at various times up to 24 h in the previous study (26), whereas in this study, blood samples were obtained over a 36-h period following drug administration. In addition, different tablet formulations [famciclovir tablet used in this study from Sichuan Baicao Biopharmaceutical Co., Ltd., China; those used in the previous study from Novartis Pharmaceuticals Corp, East Hanover, NJ, USA (26)] may also possibly affect the *C*_max_ and AUC when sadministered orally. The difference of lower *C*_max_ (7.57 ± 0.56 μg/mL compared to 18.6 ± 6.5 μg/mL in the previous study) and AUC (25.82 ± 5.51 h μg mL^−1^ compared to 41.1 ± 7.6 h μg mL^−1^ in the previous study) after intravenous infusion of 10 mg/kg penciclovir was most likely attributable to differences in blood infusion/collection method and location. In the previous study (26); one side, jugular vein infusion and contralateral jugular vein blood collection were used when studying bioavailability after intravenous administration of penciclovir, with “central venous blood” exhibiting a higher peak value and an earlier peak time. Conversely, we chose one side radial vein infusion and contralateral radial vein blood collection. In this study, the fully mixed peripheral venous blood represents the blood that has been metabolized by the majority of the tissues throughout the body, characterized by a lower peak and a slower curve. We used blood samples from the infusion/blood collection models of “radial vein radial vein” because they may more stably reflect the equilibrium concentration in systemic tissues, better reflect the long-term efficacy of drugs or the exposure level of peripheral tissues, and have less stimulation on experimental animals. Due to fundamental physiological and circulatory pathway differences between the two infusion/blood collection models of “radial vein radial vein” and “jugular vein jugular vein,” there were significant differences in the accuracy and representativeness of PK obtained, and further research on the sample differences between these two methods was needed. Our multiple oral administration period was 14 days, which was longer than the previous relevant reports (3 days) (24, 25), making the data comparison less meaningful.

From the pharmacokinetic data of multiple oral administrations of 62.5 mg/kg famciclovir tablets twice a day for 14 consecutive days, the concentration of the drug fluctuates violently in steady state, which may result in *C*_min,ss_ being lower than the minimum effective concentration. Therefore, we need to combine pharmacological experiments to observe whether administering 62.5 mg/kg famciclovir tablets twice a day can meet clinical treatment needs. If not, shortening the dosing interval, switching to sustained-release formulations, or increasing the dosage could increase *C*_min,ss_.

Throughout the entire experiment, all the tested cats were in good condition, and there were no deaths or illnesses. Better still, famciclovir tablets have almost no accumulation in cats after multiple oral administrations, with both accumulation factors (Rac_*C*_max_ and Rac_AUC) close to 1, indicating no risk of accumulated poisoning. Among the adverse events caused by the clinical use of famciclovir in human medicine, headache, nausea, and diarrhea are the most common, with mild to moderate intensity, lasting 1–2 days, and can self-heal without treatment (23). Therefore, further research is needed on the safety and adverse reactions of famciclovir tablets in cats.

FVR caused by FHV-1 is a common and serious infectious upper respiratory disease, whose clinical signs consist of conjunctivitis, ocular lesions, nasal discharge, sneezing, dyspnea, fever, lethargy, inappetence, and oral and skin ulcers in cats. Symptomatic therapy is important in the management of cats with FVR, and so the combined use of multiple therapeutic drugs has alleviated symptoms and accelerated recovery. Famciclovir, as an effective drug for systemic therapy in cats with FHV-1-related clinical signs, can be used in combination with various medications to facilitate the treatment of FVR, such as Ganciclovir Eye Drops used locally, L-lysine, immunoglobulins, doxycycline, and interferon (30–32).

In summary, after oral administration in cats, the absorption and exposure of famciclovir tablets increase with increasing dose; however, the rate of increase in exposure gradually decreases, showing overall non-linear pharmacokinetic characteristics, which is consistent with literature reports. After a single oral administration of doses ranging from 62.5 to 93.75 mg/kg, there was no significant increase in *C*_max_ and AUC, indicating that increasing the dosage does not contribute to therapeutic efficacy and may even increase adverse drug reactions. The initial safety of the famciclovir tablet in cats after multiple oral administrations, with 62.5 mg/kg administered twice daily for 14 consecutive days, had been demonstrated. Gender had little effect on the oral absorption ability of famciclovir tablets in cats. This study provides a scientific basis for the clinical dosage and duration of treatment of FVR with famciclovir tablets.

## Data Availability

The original contributions presented in the study are included in the article/Supplementary material, further inquiries can be directed to the corresponding author.
