# Supplementary material for: Non-linear pharmacokinetics of penciclovir in healthy cats after single and multiple oral administration of famciclovir
Source: Front Vet Sci. 2025 Dec 1;12:1695827. doi: 10.3389/fvets.2025.1695827 (PMC12704320; doi:10.3389/fvets.2025.1695827)
Supplement: Supplementary file 1 [file Data_Sheet_1.docx]

Supplementary Material


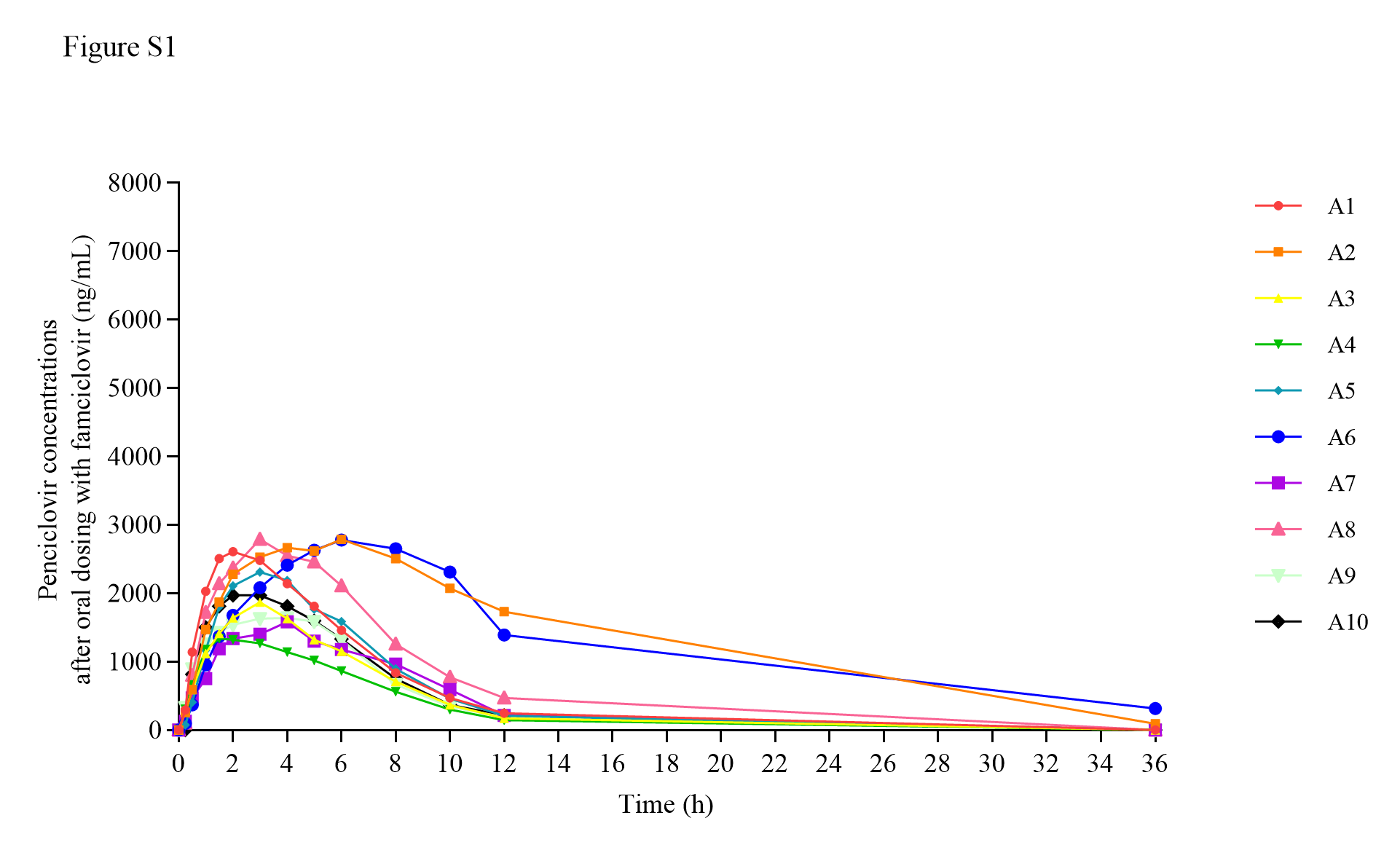


Figure S1. Plasma concentration time curve of penciclovir after oral administration of 15.625 mg/kg famciclovir in the test cat (Group A).


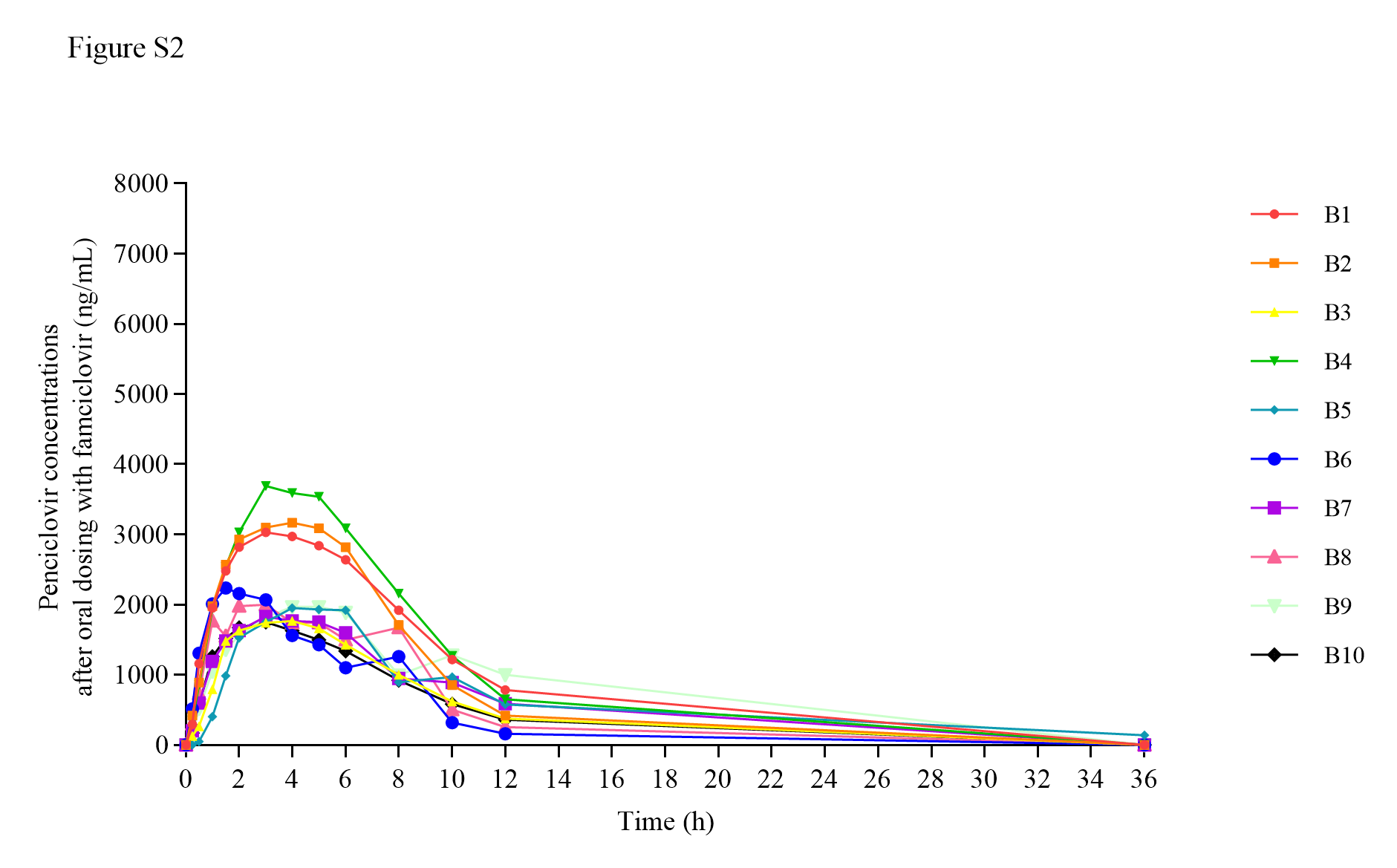


Figure S2. Plasma concentration time curve of penciclovir after oral administration of 31.25 mg/kg famciclovir in the test cat (Group B).


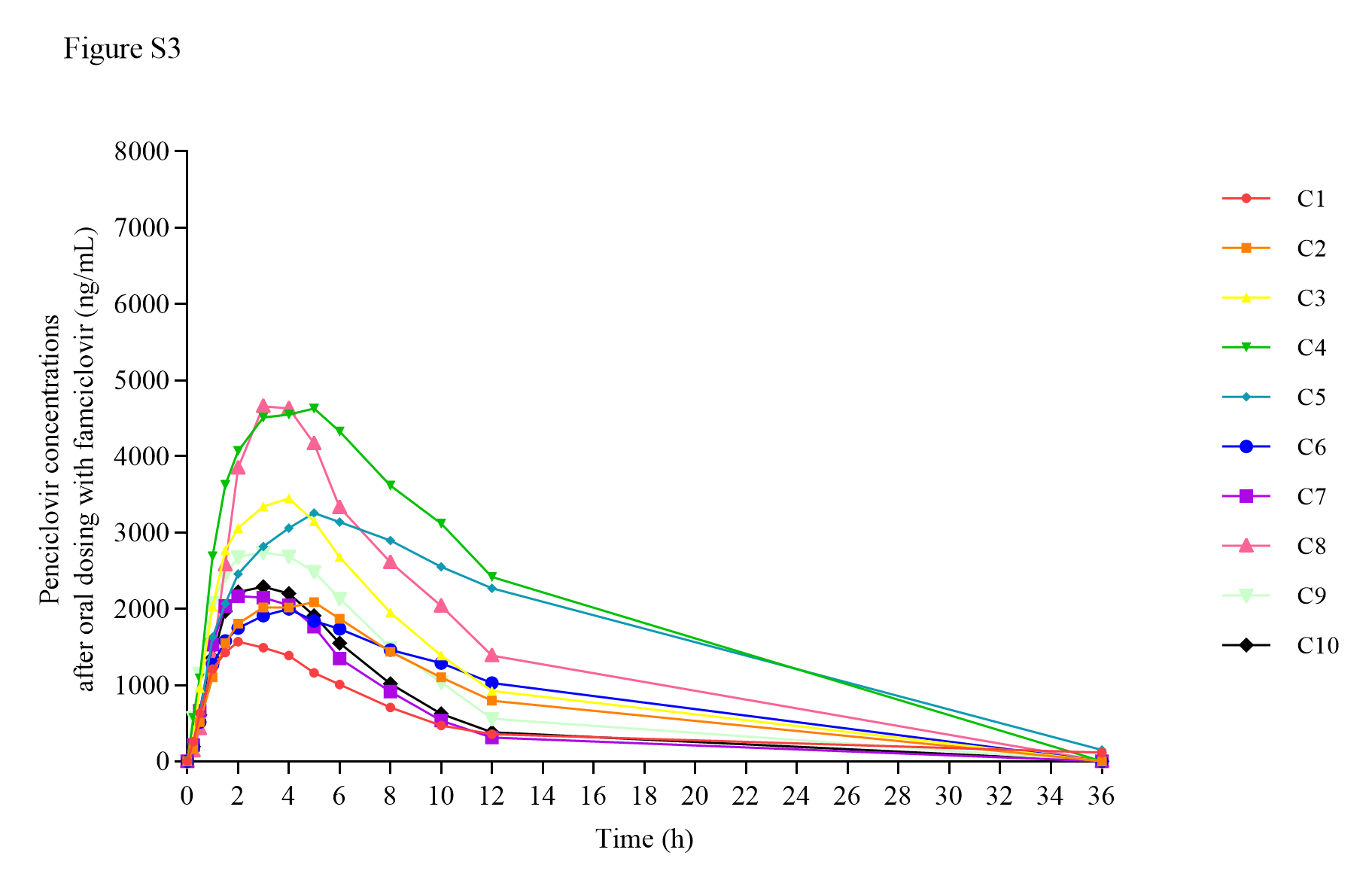


Figure S3. Plasma concentration time curve of penciclovir after oral administration of 62.5 mg/kg famciclovir in the test cat (Group C).


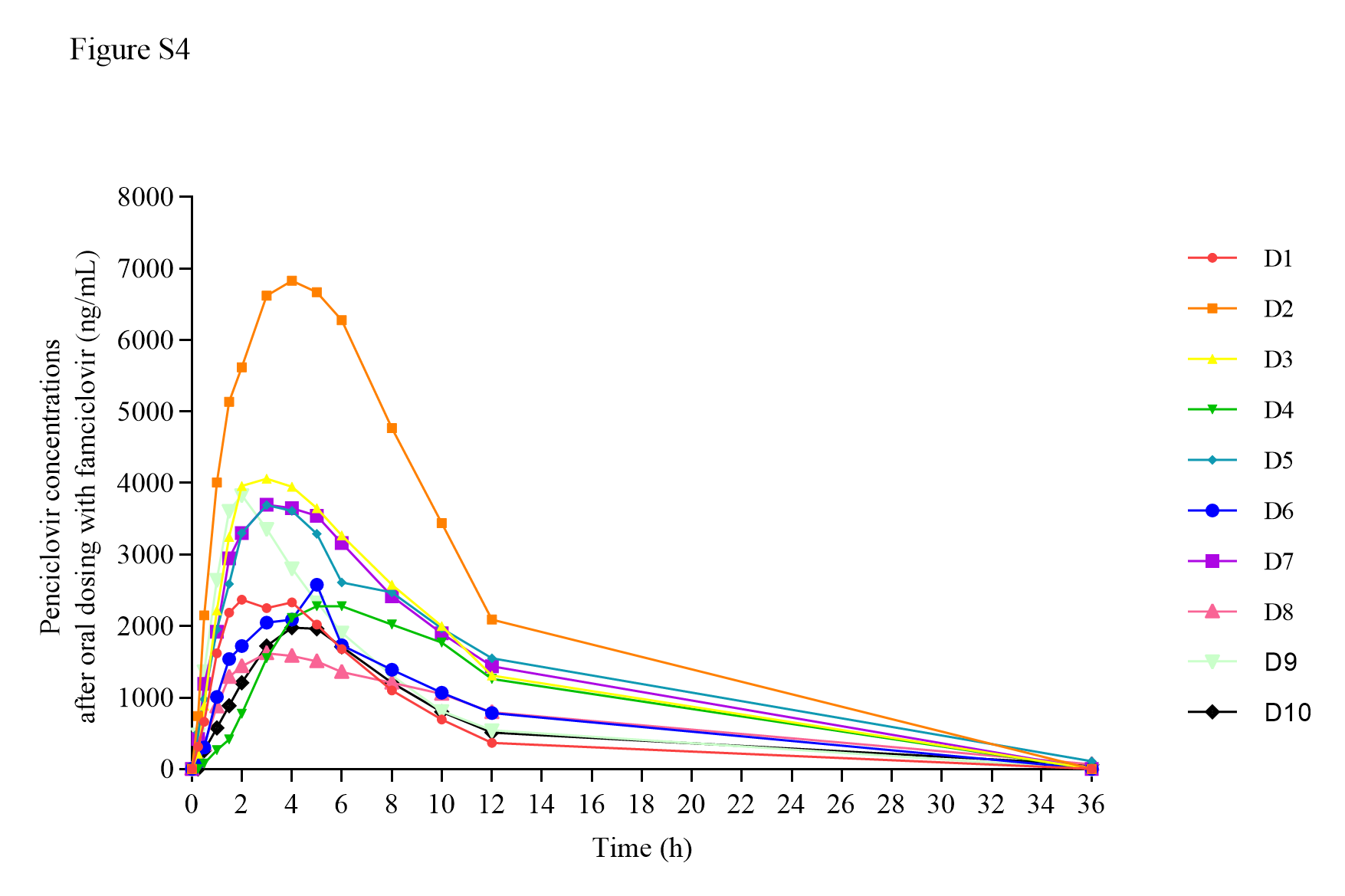


Figure S4. Plasma concentration time curve of penciclovir after oral administration of 93.75 mg/kg famciclovir in the test cat (Group D).

Table S1. Interpretation of pharmacokinetic parameters in single dose studies

| PK | Interpretation |
| --- | --- |
| C_max_ | The maximum concentration (peak concentration) of the drug reached after administration, expressed as the actual observed value |
| AUC_0-t_ | The area under the drug concentration time curve from zero to the last measurable drug concentration time point, where t is the time of the last quantifiable sample concentration, is calculated using the linear logarithmic trapezoidal method of Phoenix Winnonlin software.  $\mathrm{AUC}_{0\to t}=\sum_{i=1}^{n} \frac{C_{i}+C_{i-1}}{2}\left( t_{i}-t_{i-1} \right)$ |
| AUC_0-∞_ | Calculate the area under the drug concentration time curve from zero to infinity using the following formula, where C_t_ is the last measurable drug concentration:  $A\text{U}\text{C}_{0-\infty}\text{=}\text{AUC}_{0-t}\text{+}\frac{\text{C}_{\text{t}}}{\text{λ}_{\text{z}}}$ |
| T_max_ | The time to reach C_max_ after administration, and the actual observed value represents |
| t_1/2_ | End elimination half-life: t_1/2_ = ln2 / λ_z_ |
| λ_z_ | To eliminate the rate constant, the slope of the straight line at the end of the logarithmic blood drug concentration time curve is used. In Phoenix Winnonlin software, the concentration values of the non-zero concentration points up to C_max_ at the end 3, 4, and 5 points are used in sequence. The logarithm is taken for regression fitting, and the Adjusted R^2^ for each fitting is calculated. Finally, the elimination slope corresponding to the concentration point with the maximum Adjusted R^2^ is selected. Adjusted R^2^ is calculated according to the following formula, where r is the correlation coefficient for each fitting, and n is the number of endpoint concentration points used for each fitting:  $\mathrm{Adjusted}R^{2}=1-\frac{\left( 1-r^{2} \right)\times\left( n-1 \right)}{\left( n-2 \right)}$ |
| AUC__%Extrap_ | The percentage of extrapolated AUC to the total AUC is calculated according to the following formula:  $A\text{U}\text{C}_{\_\%Extrap}\text{=}\frac{A\text{U}\text{C}_{0-\infty}-\text{AUC}_{0-t}}{A\text{U}\text{C}_{0-\infty}}\times100\%$ |
| CL/F | The apparent clearance rate is calculated according to the following formula:  CL/F = Dose / AUC_0-∞_ |
| V_z_/F | The apparent distribution volume is calculated according to the following formula:  V/F = CL/F / λ_z_ |
| MRT_0-t_ | The average residence time from zero to the last measurable drug concentration time point is the first moment of the probability density constant f (t) of the drug's residence time in the body. It is the average time that all drug molecules remain in the body, calculated according to the following formula:  $\mathrm{MRT}_{0-t}\text{=}{\mathrm{AUMC}_{0-t}}/{\mathrm{AUC}_{0-t}}$  $\mathrm{AUMC}_{0\to t}=\sum_{i=1}^{n} \frac{{t_{i}C}_{i}+{t_{i-1}C}_{i-1}}{2}\left( t_{i}-t_{i-1} \right)$ |
| MRT_0-∞_ | Calculate the average residence time from zero to infinity using the following formula:  $\mathrm{MRT}_{0-\infty}\text{=}{\mathrm{AUMC}_{0-\infty}}/{\mathrm{AUC}_{0-\infty}}$  $\mathrm{AUMC}_{0\to\infty}=\sum_{i=1}^{n} \frac{{t_{i}C}_{i}+{t_{i-1}C}_{i-1}}{2}\left( t_{i}-t_{i-1} \right)+\left( {C_{t}}/{\lambda_{z}^{2}}+{{t\cdot C}_{t}}/{\lambda_{z}} \right)$ |

Table S2. Interpretation of pharmacokinetic parameters in multiple administration studies

| PK | Interpretation |
| --- | --- |
| C_max,D1_ | The maximum concentration (peak concentration) of the drug reached after first administration, expressed as the actual observed value. |
| T_max,D1_ | The time to reach C_max,D1_ after administration will be determined based on the measured values. |
| C_max,ss_ | The maximum blood drug concentration at steady state will be determined based on the measured values. |
| C_min,ss_ | The minimum blood drug concentration at steady state will be determined based on the measured values. |
| C_av,ss_ | Calculate the average blood drug concentration within the dosing interval at steady state using the following formula，τ is dosing interval：  C_av,ss_ = AUC_0-τ,ss_ / τ |
| T_max,ss_ | The peak time of blood drug concentration at steady state will be determined based on the measured values. |
| AUC_0-τ,D1_ | After the first administration on the first day, calculate the area under the drug concentration time curve until τ hours after the start of treatment using the trapezoidal area method. |
| AUC_0-τ,ss_ | After reaching steady state, calculate the area under the drug concentration time curve using the trapezoidal area method until τ hours after the start of treatment. |
| t_1/2_ | The half-life of terminal elimination is calculated according to the following formula:  t_1/2_ = ln2 / λ_z_ |
| CL_ss_/F | Steady state apparent clearance rate, calculated according to the following formula:  CL_ss_/F = Dose/AUC_0-τ_ |
| V_ss_/F | Calculate the steady-state apparent distribution volume according to the following formula:  V_ss_/F = Dose /(AUC_0-τ_∙λ_z_) |
| DF | The fluctuation coefficient, the ratio of the difference between the steady-state maximum blood drug concentration and the steady-state minimum blood drug concentration to the average steady-state blood drug concentration, is calculated according to the following formula:  $\text{DF = }\frac{C_{max, ss}-C_{min,ss}}{\bar{C_{\mathrm{ss}}}}\times100\%$ |
| Rac_C_max_ | Calculate the accumulation index based on C_max_ using the following formula:  Rac_C_max_ = C_max,ss_ / C_max,D1_ |
| Rac_AUC | Calculate the accumulation index based on AUC_0- τ_ using the following formula:  Rac_AUC_0-τ_ = AUC_0-τ,ss_ / AUC_0-τ,D1_ |

Table S3. Statistics on different dosage differences in the pharmacokinetic parameters (C_max_, AUC_0-t_, AUC_0-∞_) after single administration in cats.

| PK | Dosage Groups | | | p-value (two-tailed) |
| --- | --- | --- | --- | --- |
| C_max_ | 15.625mg/kg | vs | 31.25mg/kg | 0.0023** |
|  | 15.625mg/kg | vs | 62.5mg/kg | <0.0001**** |
|  | 15.626mg/kg | vs | 93.75mg/kg | <0.0002**** |
|  | 31.25mg/kg | vs | 62.5mg/kg | 0.0088** |
|  | 31.26mg/kg | vs | 93.75mg/kg | 0.0021** |
|  | 62.5mg/kg | vs | 93.75mg/kg | >0.9999 |
| AUC_0-t_ | 15.625mg/kg | vs | 31.25mg/kg | 0.0364* |
|  | 15.625mg/kg | vs | 62.5mg/kg | 0.0056** |
|  | 15.626mg/kg | vs | 93.75mg/kg | 0.0024** |
|  | 31.25mg/kg | vs | 62.5mg/kg | 0.059 |
|  | 31.26mg/kg | vs | 93.75mg/kg | 0.0044** |
|  | 62.5mg/kg | vs | 93.75mg/kg | 0.2459 |
| AUC_0-∞_ | 15.625mg/kg | vs | 31.25mg/kg | 0.0496* |
|  | 15.625mg/kg | vs | 62.5mg/kg | 0.0094** |
|  | 15.626mg/kg | vs | 93.75mg/kg | 0.0034** |
|  | 31.25mg/kg | vs | 62.5mg/kg | 0.0959 |
|  | 31.26mg/kg | vs | 93.75mg/kg | 0.0062** |
|  | 62.5mg/kg | vs | 93.75mg/kg | 0.2055 |

* represented significantly different (P < 0.05), ** represented extremely significantly different (P < 0.01), ****represented very significantly different (P < 0.0001).

Table S4. Statistics on gender differences in the pharmacokinetic parameters of penciclovir in plasma after single/multiple administration and intravenous infusion in cats.

| Phase Ⅰ | Dosage Groups | Progressive significance test p-value (two-tailed) | | | | | | | | | | | | | | | | | | | | | | |
| --- | --- | --- | --- | --- | --- | --- | --- | --- | --- | --- | --- | --- | --- | --- | --- | --- | --- | --- | --- | --- | --- | --- | --- | --- |
|  |  | λz | | t_1/2_ | | | T_max_ | | C_max_ | | | AUC_0-t_ | | AUC_0-∞_ | | Vz/F | | Cl/F | | | MRT_0-t_ | | MRT_0-∞_ | |
| Single Administration | 15.625mg/kg | 0.602 | | 0.602 | | | 0.337 | | 1.000 | | | 0.917 | | 0.754 | | 0.465 | | 0.602 | | | 0.347 | | 0.175 | |
|  | 31.25  mg/kg | 0.754 | | 0.754 | | | 0.166 | | 0.251 | | | 0.076 | | 0.117 | | 0.347 | | 0.076 | | | 0.602 | | 0.602 | |
|  | 62.5  mg/kg | 0.117 | | 0.117 | | | 0.106 | | 0.917 | | | 0.251 | | 0.251 | | 0.754 | | 0.117 | | | 0.047* | | 0.117 | |
|  | 93.75  mg/kg | 0.602 | | 0.602 | | | 1.000 | | 0.347 | | | 0.465 | | 0.251 | | 0.251 | | 0.251 | | | 0.602 | | 0.602 | |
| Phase Ⅱ | Dosage Groups | Progressive significance test p-value (two-tailed) | | | | | | | | | | | | | | | | | | | | | | |
|  |  | t_1/2_ | T_max,D1_ | | C_max,D1_ | T_max,ss_ | | C_max,ss_ | | C_min,ss_ | C_av,ss_ | | AUC_0-12,D1_ | | AUC_0-12,ss_ | | V_ss_/F | | CL_ss_/F | DF | | Rac_C_max_ | | Rac_AUC |
| Multiple Administration | 62.5  mg/kg | 0.465 | 0.700 | | 0.117 | 0.654 | | 0.175 | | 0.094 | 0.175 | | 0.754 | | 0.175 | | 0.117 | | 0.251 | 0.251 | | 0.832 | | 0.465 |
| Phase Ⅲ | Dosage Groups | Progressive significance test p-value (two-tailed) | | | | | | | | | | | | | | | | | | | | | | |
|  |  | λz | | t_1/2_ | | | T_max_ | | C_max_ | | | AUC_0-t_ | | AUC_0-∞_ | | Vz/F | | Cl/F | | | MRT_0-t_ | | MRT_0-∞_ | |
| Intravenous Infusion | 10 mg/kg | 0.602 | | 0.602 | | | 0.134 | | 0.251 | | | 0.917 | | 0.917 | | 0.016* | | 0.465 | | | 0.602 | | 0.602 | |

* represented significantly different (P < 0.05)
