# Supplementary material for: Non-linear pharmacokinetics of penciclovir in healthy cats after single and multiple oral administration of famciclovir
Source: Front Vet Sci. 2025 Dec 1;12:1695827. doi: 10.3389/fvets.2025.1695827 (PMC12704320; doi:10.3389/fvets.2025.1695827)
Supplement: Supplementary file 2 [file Table_1.docx]

Table S1. Interpretation of pharmacokinetic parameters in single dose studies.

| PK | Interpretation |
| --- | --- |
| C_max_ | The maximum concentration (peak concentration) of the drug reached after administration, expressed as the actual observed value |
| AUC_0-t_ | The area under the drug concentration time curve from zero to the last measurable drug concentration time point, where t is the time of the last quantifiable sample concentration, is calculated using the linear logarithmic trapezoidal method of Phoenix Winnonlin software.  $\mathrm{AUC}_{0\to t}=\sum_{i=1}^{n} \frac{C_{i}+C_{i-1}}{2}\left( t_{i}-t_{i-1} \right)$ |
| AUC_0-∞_ | Calculate the area under the drug concentration time curve from zero to infinity using the following formula, where C_t_ is the last measurable drug concentration:  $A\text{U}\text{C}_{0-\infty}\text{=}\text{AUC}_{0-t}\text{+}\frac{\text{C}_{\text{t}}}{\text{λ}_{\text{z}}}$ |
| T_max_ | The time to reach C_max_ after administration, and the actual observed value represents |
| t_1/2_ | End elimination half-life: t_1/2_ = ln2 / λ_z_ |
| λ_z_ | To eliminate the rate constant, the slope of the straight line at the end of the logarithmic blood drug concentration time curve is used. In Phoenix Winnonlin software, the concentration values of the non-zero concentration points up to C_max_ at the end 3, 4, and 5 points are used in sequence. The logarithm is taken for regression fitting, and the Adjusted R^2^ for each fitting is calculated. Finally, the elimination slope corresponding to the concentration point with the maximum Adjusted R^2^ is selected. Adjusted R^2^ is calculated according to the following formula, where r is the correlation coefficient for each fitting, and n is the number of endpoint concentration points used for each fitting:  $\mathrm{Adjusted}R^{2}=1-\frac{\left( 1-r^{2} \right)\times\left( n-1 \right)}{\left( n-2 \right)}$ |
| AUC__%Extrap_ | The percentage of extrapolated AUC to the total AUC is calculated according to the following formula:  $A\text{U}\text{C}_{\_\%Extrap}\text{=}\frac{A\text{U}\text{C}_{0-\infty}-\text{AUC}_{0-t}}{A\text{U}\text{C}_{0-\infty}}\times100\%$ |
| CL/F | The apparent clearance rate is calculated according to the following formula:  CL/F = Dose / AUC_0-∞_ |
| V_z_/F | The apparent distribution volume is calculated according to the following formula:  V/F = CL/F / λ_z_ |
| MRT_0-t_ | The average residence time from zero to the last measurable drug concentration time point is the first moment of the probability density constant f (t) of the drug's residence time in the body. It is the average time that all drug molecules remain in the body, calculated according to the following formula:  $\mathrm{MRT}_{0-t}\text{=}{\mathrm{AUMC}_{0-t}}/{\mathrm{AUC}_{0-t}}$  $\mathrm{AUMC}_{0\to t}=\sum_{i=1}^{n} \frac{{t_{i}C}_{i}+{t_{i-1}C}_{i-1}}{2}\left( t_{i}-t_{i-1} \right)$ |
| MRT_0-∞_ | Calculate the average residence time from zero to infinity using the following formula:  $\mathrm{MRT}_{0-\infty}\text{=}{\mathrm{AUMC}_{0-\infty}}/{\mathrm{AUC}_{0-\infty}}$  $\mathrm{AUMC}_{0\to\infty}=\sum_{i=1}^{n} \frac{{t_{i}C}_{i}+{t_{i-1}C}_{i-1}}{2}\left( t_{i}-t_{i-1} \right)+\left( {C_{t}}/{\lambda_{z}^{2}}+{{t\cdot C}_{t}}/{\lambda_{z}} \right)$ |
