# Supplementary material for: Non-linear pharmacokinetics of penciclovir in healthy cats after single and multiple oral administration of famciclovir
Source: Front Vet Sci. 2025 Dec 1;12:1695827. doi: 10.3389/fvets.2025.1695827 (PMC12704320; doi:10.3389/fvets.2025.1695827)
Supplement: Supplementary file 3 [file Table_2.docx]

Table S2. Interpretation of pharmacokinetic parameters in multiple administration studies.

| PK | Interpretation |
| --- | --- |
| C_max,D1_ | The maximum concentration (peak concentration) of the drug reached after first administration, expressed as the actual observed value. |
| T_max,D1_ | The time to reach C_max,D1_ after administration will be determined based on the measured values. |
| C_max,ss_ | The maximum blood drug concentration at steady state will be determined based on the measured values. |
| C_min,ss_ | The minimum blood drug concentration at steady state will be determined based on the measured values. |
| C_av,ss_ | Calculate the average blood drug concentration within the dosing interval at steady state using the following formula，τ is dosing interval：  C_av,ss_ = AUC_0-τ,ss_ / τ |
| T_max,ss_ | The peak time of blood drug concentration at steady state will be determined based on the measured values. |
| AUC_0-τ,D1_ | After the first administration on the first day, calculate the area under the drug concentration time curve until τ hours after the start of treatment using the trapezoidal area method. |
| AUC_0-τ,ss_ | After reaching steady state, calculate the area under the drug concentration time curve using the trapezoidal area method until τ hours after the start of treatment. |
| t_1/2_ | The half-life of terminal elimination is calculated according to the following formula:  t_1/2_ = ln2 / λ_z_ |
| CL_ss_/F | Steady state apparent clearance rate, calculated according to the following formula:  CL_ss_/F = Dose/AUC_0-τ_ |
| V_ss_/F | Calculate the steady-state apparent distribution volume according to the following formula:  V_ss_/F = Dose /(AUC_0-τ_∙λ_z_) |
| DF | The fluctuation coefficient, the ratio of the difference between the steady-state maximum blood drug concentration and the steady-state minimum blood drug concentration to the average steady-state blood drug concentration, is calculated according to the following formula:  $\text{DF = }\frac{C_{max, ss}-C_{min,ss}}{\bar{C_{\mathrm{ss}}}}\times100\%$ |
| Rac_C_max_ | Calculate the accumulation index based on C_max_ using the following formula:  Rac_C_max_ = C_max,ss_ / C_max,D1_ |
| Rac_AUC | Calculate the accumulation index based on AUC_0- τ_ using the following formula:  Rac_AUC_0-τ_ = AUC_0-τ,ss_ / AUC_0-τ,D1_ |
