# Supplementary material for: Non-linear pharmacokinetics of penciclovir in healthy cats after single and multiple oral administration of famciclovir
Source: Front Vet Sci. 2025 Dec 1;12:1695827. doi: 10.3389/fvets.2025.1695827 (PMC12704320; doi:10.3389/fvets.2025.1695827)
Supplement: Supplementary file 4 [file Table_3.docx]

Table S3. Statistics on different dosage differences in the pharmacokinetic parameters (C_max_, AUC_0-t_, AUC_0-∞_) after single administration in cats.

| PK | Dosage Groups | | | P-Value (two-tailed) |
| --- | --- | --- | --- | --- |
| C_max_ | 15.625mg/kg | vs | 31.25mg/kg | 0.0023** |
|  | 15.625mg/kg | vs | 62.5mg/kg | <0.0001**** |
|  | 15.626mg/kg | vs | 93.75mg/kg | <0.0002**** |
|  | 31.25mg/kg | vs | 62.5mg/kg | 0.0088** |
|  | 31.26mg/kg | vs | 93.75mg/kg | 0.0021** |
|  | 62.5mg/kg | vs | 93.75mg/kg | >0.9999 |
| AUC_0-t_ | 15.625mg/kg | vs | 31.25mg/kg | 0.0364* |
|  | 15.625mg/kg | vs | 62.5mg/kg | 0.0056** |
|  | 15.626mg/kg | vs | 93.75mg/kg | 0.0024** |
|  | 31.25mg/kg | vs | 62.5mg/kg | 0.059 |
|  | 31.26mg/kg | vs | 93.75mg/kg | 0.0044** |
|  | 62.5mg/kg | vs | 93.75mg/kg | 0.2459 |
| AUC_0-∞_ | 15.625mg/kg | vs | 31.25mg/kg | 0.0496* |
|  | 15.625mg/kg | vs | 62.5mg/kg | 0.0094** |
|  | 15.626mg/kg | vs | 93.75mg/kg | 0.0034** |
|  | 31.25mg/kg | vs | 62.5mg/kg | 0.0959 |
|  | 31.26mg/kg | vs | 93.75mg/kg | 0.0062** |
|  | 62.5mg/kg | vs | 93.75mg/kg | 0.2055 |

* represented significantly different (P < 0.05)，** represented extremely significantly different (P < 0.01)， ****represented very significantly different (P < 0.0001).
