# Supplementary material for: Non-linear pharmacokinetics of penciclovir in healthy cats after single and multiple oral administration of famciclovir
Source: Front Vet Sci. 2025 Dec 1;12:1695827. doi: 10.3389/fvets.2025.1695827 (PMC12704320; doi:10.3389/fvets.2025.1695827)
Supplement: Supplementary file 5 [file Table_4.docx]

Table S4. Statistics on gender differences in the pharmacokinetic parameters of penciclovir in plasma after single/multiple administration and intravenous infusion in cats.

| Phase Ⅰ | Dosage Groups | Progressive significance test p-value (two-tailed) | | | | | | | | | | | | | | | | | | | | | | |
| --- | --- | --- | --- | --- | --- | --- | --- | --- | --- | --- | --- | --- | --- | --- | --- | --- | --- | --- | --- | --- | --- | --- | --- | --- |
|  |  | λz | | t_1/2_ | | | T_max_ | | C_max_ | | | AUC_0-t_ | | AUC_0-∞_ | | Vz/F | | Cl/F | | | MRT_0-t_ | | MRT_0-∞_ | |
| Single Administration | 15.625mg/kg | 0.602 | | 0.602 | | | 0.337 | | 1.000 | | | 0.917 | | 0.754 | | 0.465 | | 0.602 | | | 0.347 | | 0.175 | |
|  | 31.25  mg/kg | 0.754 | | 0.754 | | | 0.166 | | 0.251 | | | 0.076 | | 0.117 | | 0.347 | | 0.076 | | | 0.602 | | 0.602 | |
|  | 62.5  mg/kg | 0.117 | | 0.117 | | | 0.106 | | 0.917 | | | 0.251 | | 0.251 | | 0.754 | | 0.117 | | | 0.047* | | 0.117 | |
|  | 93.75  mg/kg | 0.602 | | 0.602 | | | 1.000 | | 0.347 | | | 0.465 | | 0.251 | | 0.251 | | 0.251 | | | 0.602 | | 0.602 | |
| Phase Ⅱ | Dosage Groups | Progressive significance test p-value (two-tailed) | | | | | | | | | | | | | | | | | | | | | | |
|  |  | t_1/2_ | T_max,D1_ | | C_max,D1_ | T_max,ss_ | | C_max,ss_ | | C_min,ss_ | C_av,ss_ | | AUC_0-12,D1_ | | AUC_0-12,ss_ | | V_ss_/F | | CL_ss_/F | DF | | Rac_C_max_ | | Rac_AUC |
| Multiple Administration | 62.5  mg/kg | 0.465 | 0.700 | | 0.117 | 0.654 | | 0.175 | | 0.094 | 0.175 | | 0.754 | | 0.175 | | 0.117 | | 0.251 | 0.251 | | 0.832 | | 0.465 |
| Phase Ⅲ | Dosage Groups | Progressive significance test p-value (two-tailed) | | | | | | | | | | | | | | | | | | | | | | |
|  |  | λz | | t_1/2_ | | | T_max_ | | C_max_ | | | AUC_0-t_ | | AUC_0-∞_ | | Vz/F | | Cl/F | | | MRT_0-t_ | | MRT_0-∞_ | |
| Intravenous Infusion | 10 mg/kg | 0.602 | | 0.602 | | | 0.134 | | 0.251 | | | 0.917 | | 0.917 | | 0.016* | | 0.465 | | | 0.602 | | 0.602 | |

* represented significantly different (P < 0.05)
